# A Novel Cogu-like Virus Identified in Wine Grapes

**DOI:** 10.3390/v17091175

**Published:** 2025-08-28

**Authors:** Jennifer Dahan, Gardenia E. Orellana, Edison Reyes-Proaño, Jungmin Lee, Alexander V. Karasev

**Affiliations:** 1 Department of Entomology, Plant Pathology and Nematology, University of Idaho, Moscow, ID 83844-2329, USA; jdahan@uidaho.edu (J.D.); gardeniao@uidaho.edu (G.E.O.); edison@uidaho.edu (E.R.-P.); 2 Independent Researcher, Corvallis, OR 97330, USA; jungminr2025@gmail.com

**Keywords:** grapevine-associated cogu-like Idaho virus, Chardonnay, virus evolution

## Abstract

A new negative-strand RNA virus was identified in grapevines from a 38-year-old ‘Chardonnay’ block in Idaho through high-throughput sequencing (HTS) of total RNA. This virus was tentatively named grapevine-associated cogu-like Idaho virus (GaCLIdV). GaCLIdV has three negative-sense, single-stranded RNA genome segments of ca. 7 kb, 1.9 kb, and 1.3 kb, encoding L protein (RNA-dependent RNA polymerase, RdRP), a movement protein (MP), and a nucleocapsid protein (NC), respectively, identified based on pair-wise comparisons with other cogu- and cogu-like viruses. In phylogenetic analysis based on the RdRP, GaCLIdV grouped within the family *Phenuiviridae* and was placed in a lineage of plant-infecting phenuiviruses as a sister clade of the genus *Laulavirus*, clustering most closely with switchgrass phenui-like virus 1 (SgPLV-1) and more distantly related to grapevine-associated cogu-like viruses from the *Laulavirus* and *Coguvirus* clades. Both GaCLIdV and SgPhLV-1 are proposed to form a new genus, Switvirus, within the family *Phenuiviridae*. The presence of GaCLIdV in the original ‘Chardonnay’ samples was confirmed by RT-PCR amplification and Sanger sequencing. This new virus was found in five wine grape cultivars and in six vineyards sampled in Idaho and in Oregon during the 2020–2024 seasons. GaCLIdV may have contributed to the decline observed in the old ‘Chardonnay’ block, although the role of the virus in symptom development awaits further investigation.

## 1. Introduction

More than 100 viruses have been reported to infect grapevines (*Vitis* spp.), with positive-sense (+), single-stranded (ss) RNA viruses dominating the global grapevine virome, which at the moment includes not more than 4% of negative-sense (-) ssRNA viruses [1,2]. Prior to 2019, there was an absence of known (-) ssRNA genomes among grapevine viruses [1,3]. However, routine applications of high-throughput sequencing (HTS) methods greatly facilitated the discovery of new viruses, and one varicosavirus (family *Rhabdoviridae*) and one emaravirus (family *Fimoviridae*) have been reported since then [4]. Additionally, at least six (-) ssRNA viruses were found since 2019 in grapevine, classified as belonging to the family *Phenuiviridae* [3,5,6]. These phenuiviruses have tri-partite genomes and are assigned to the genera *Bocivirus*, *Laulavirus*, and *Rubodvirus* [7].

*Phenuiviridae* is a large virus family that currently includes 23 genera and 159 species approved by the International Committee on Taxonomy of Viruses (ICTV) with segmented negative-sense or ambisense RNA genomes [7]. Phenuiviruses infecting animals have enveloped spherical or pleiomorphic virions, 80–120 nm in diameter, with filamentous nucleocapsids inside, but virions representing non-enveloped filamentous nucleocapsids were reported for plant-infecting phenuiviruses [7,8]. Seven genera of the family *Phenuiviridae* comprise plant-infecting or plant-associated viruses: *Bocivirus*, *Coguvirus*, *Goukovirus*, *Laulavirus*, *Mechlorovirus*, *Rubodvirus*, and *Tenuivirus*. Although insect transmission is characteristic of tenuiviruses [8], for most of the plant-infecting phenuiviruses, including grapevine-infecting phenuiviruses, the only known mode of transmission is by grafting [3,7].

Commercial production of wine started in Idaho in the early 1970s, and currently most of the classic red- and white-berried varieties are grown across the state. In 2025, there were 65 wineries in the state of Idaho, growing wine grapes on approximately 550 hectares. Two main American viticulture areas (AVAs) have been established in the state, one in the south-west (Snake River Valley AVA) and one in the north (Lewis–Clark Valley AVA). Grapevine viruses are recognized as one of the limiting factors in wine grape production in Idaho, and systematic testing for viruses was initiated in 2008. Since 2009, multiple viruses and viroids were reported from grapevines in the state, including grapevine leafroll-associated virus 3 (GLRaV-3) [9,10], grapevine fleck virus (GFkV) [11], grapevine red blotch virus (GRBV) [12], grapevine rupestris vein feathering virus (GRVFV) [13], grapevine-associated tymo-like virus (GaTLV) [14], grapevine rupestris stem pitting-associated virus (GRSPaV) [15], three endornaviruses [16], and four grapevine-associated viroids [10,17]. While many of these described viruses could not be associated with the main disease syndromes plaguing wine grapes, at least two of these viruses, GLRaV-3 and GRBV, were found to negatively impact grape quality [18,19].

Here, we report the discovery of a new cogu-like virus in Idaho grapevines, in several cultivars. This new virus was discovered through the application of HTS, and the subsequent validation and confirmation of its presence was achieved by conventional RT-PCR and Sanger sequencing. We also conducted a series of surveys during four seasons and assessed the prevalence of this new cogu-like virus in wine grapes in Idaho.

## 2. Materials and Methods

### 2.1. Grapevine Sampling and Sample Processing

Initial grapevine leaf and petiole samples were collected from a commercially operated vineyard in Canyon County of Idaho. A 38-year old, declining ‘Chardonnay’ block was first sampled in October 2018 in vineyard V1 and then again in September 2020. Three samples from vineyard V1, RB02, collected in 2018 [13], and RB09 and RB12, collected in 2020, were submitted to HTS analysis. The same vineyard V1 was also sampled in September 2021 and in September 2023–2024, along with five additional vineyards, V2 to V6, in Canyon County of Idaho and in Malheur County of Oregon for RT-PCR testing for various grapevine viruses. The sampling methodology followed the previously described protocol [10]. Briefly, four fully expanded leaves with complete petioles were collected per vine, from all sides of the canopy, and placed into a single plastic resealable bag labeled with the vine number and the name of the vineyard. These leaf samples were kept in a cooler with ice for 2–3 days until reaching the laboratory and then in a cold room (4 °C) until the final processing, which occurred 3–14 days after leaf collection. Petioles were cut off from the leaves and used for subsequent extraction and analysis.

### 2.2. RNA Extraction and HTS Analysis

The plant tissue was ground in plastic meshed bags (Bioreba AG, Reinach, Switzerland), total RNA was extracted using the Spectrum Plant Total RNA kit (Sigma-Aldrich, St. Louis, MO, USA) and subjected to DNase treatment using a DNase kit (QIAGEN, Germantown, MD, USA), and following a ribodepletion using the RiboMinus Plant kit for RNA-Seq (Invitrogen, Waltham, MA, USA), libraries were prepared using the Kapa RNA Hyper-Prep kit (Roche, Indianapolis, IN, USA) with NEXTflex-Unique-Dual-Index-Barcodes-Set-C (BioO Scientific, Austin, TX, USA). After a bead-based size selection the resulting libraries were multiplexed and subjected to Illumina high-throughput sequencing on a NovaSeq 6000 platform through the University of Idaho Genomics and Bioinformatics Resources Core Facility as described previously [16]. Between 30.2 and 63.0 million 250 bp paired-end reads per sample were produced. Raw reads were adapter- and quality cleaned using Trimmomatic v0.38 [20] and mapped against the *V. vinifera* L. reference genome retrieved from NCBI, using bowtie2 v2.4.4 in local mode. Unmapped paired-end reads were subjected to assembly using SPAdes v3.15.3 in RNA mode and analyses using BLASTn v2.10.1 and DIAMOND v2.0.14 [21] programs. A search for conserved protein domains was conducted using the conserved domain database (CDD) available at NCBI [22,23].

### 2.3. Nucleic Acid Extraction, RT-PCR Testing, and Sanger Sequencing

For samples collected in September 2020–2024, total RNA was extracted from grapevine leaf and petiole tissues ground in plastic meshed bags (Bioreba) following the Spectrum Plant Total RNA kit (Sigma-Aldrich, St. Louis, MO, USA) instructions. Reverse transcription was performed using 4.5 µL of the extracted RNA in a 25 µL reaction mixture that contained 5× first-strand buffer (Promega, Madison, WI, USA), 2.5 mM dNTP, 3 µM oligo dT + random hexamers, rRNasin ribonuclease inhibitor (Promega, Madison, WI, USA), and M-MLV reverse transcriptase (Promega, Madison, WI, USA). Before the reverse transcription reaction, the RNA template was incubated at 70 °C for 5 min, then the reverse transcription mix was added. The profile used included initial incubation at 25 °C for 10 min, incubation at 42 °C for 50 min, and reverse transcriptase deactivation at 70 °C for 15 min prior to PCR. All PCR products were synthesized by GreenTaq (GenScript, Piscataway, NJ, USA) in a 20 µL reaction mixture that contained 10× GreenTaq buffer, 2.5 mM dNTP, 5 µM solutions of each forward primer and reverse primer, GreenTaq, and 2 µL of cDNA template. The PCR profile consisted of denaturing at 94 °C for 2 min and 35 cycles at 94 °C for 30 s, 55–65 °C for 30 s (depending on the melting temperature of the primers used), and 72 °C for 1 to 2 min (depending on the fragment length amplified), followed by a final extension for 10 min at 72 °C. Sanger sequencing was performed on the RT-PCR fragments amplified from total RNA extracted from the infected grapevine plants as described above and followed a previously described protocol [24]. The primers used to amplify these DNA fragments are listed in Supplementary Table S1. The PCR fragments were treated with Exosap-It (ThermoFisher, Waltham, MA, USA) and submitted for sequencing to Elim Biopharmaceuticals, Inc. (Hayward, CA, USA).

### 2.4. Sequence and Phylogenetic Analysis

The phylogenetic trees for the L protein were generated based on the set of sequences used by Diaz-Lara et al. (2019) [3] with the addition of some replicase sequences from the NCBI/RefSeq *Phenuiviridae* L protein database. The tomato spotted wilt orthotospovirus reference sequence was used as an outgroup. The sequences of the replicase proteins from each virus were aligned using MAFFT in g-insi mode implemented in Geneious Prime 2023 (default parameters), and a maximum likelihood tree was inferred in IQtree 2 v2.3.5 [25] using ModelFinder for best-model selection [26] and the aBayes test for branch support estimation, available in IQtree 2 [27]. The phylogenetic tree was visualized using the ggtree package v3.16.0 in R [28].

## 3. Results

**Figure 1:**
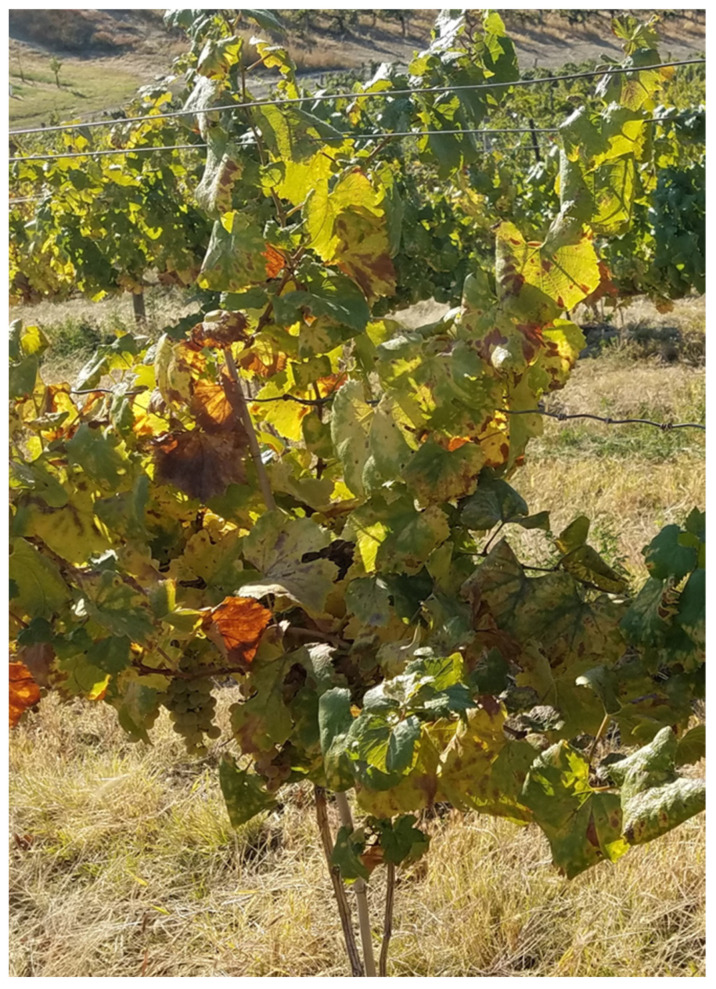
A 38-year-old ‘Chardonnay’ vine (in the first year of sampling in 2018) exhibiting decline symptoms and found positive for the grapevine-associated cogu-like Idaho virus (GaCLIdV).

In October 2018, symptoms of leaf chlorosis, yellowing, and leaf necrosis resembling premature senescence were observed in a block of 38-year-old, own-rooted ‘Chardonnay’ vines in Canyon County of Idaho (Figure 1). The owner of the vineyard described the declining productivity of the block over several years and a general unresponsiveness of the plants to fertilizing. Leaves and petioles were collected from two symptomatic ‘Chardonnay’ plants in October 2018 and then again from two plants exhibiting similar symptoms in September 2020.

When the total RNA extracted from these samples was subjected to HTS and subsequent bioinformatics analysis, one sample collected in 2018 (18RB02) and both samples collected in 2020 (RB09 and RB12) produced a sufficient number of high-quality reads (Table 1). All three samples were found infected with GLRaV-3 and GVA, while RB09 and RB12 also had GVB, GVF, GRVFV, GaTLV, and endornaviruses (see [13,14,16]).

### 3.1. Cogu-like Virus Sequences Revealed by HTS in Grapevine Leaf and Petiole Tissue

Eighteen contigs in total, of length of over 500 nt, in all three of the grapevine samples 18RB02, RB09, and RB12, were found related to cogu- and cogu-like viruses following the sequence analyses. They assembled as negative strands and ranged between 821 and 6398 nt in size. Based on pair-wise comparisons to cogu-like virus sequences in the GenBank database, several of these contigs were assumed to represent complete or nearly complete RNA1, RNA2, and RNA3 of a novel tripartite cogu-like virus, which was provisionally named grapevine-associated cogu-like Idaho virus (GaCLIdV). Following an approach of remapping and reassembling of the mapped reads against the putative segments using data from the RB09 and RB12 samples, a slight genetic diversity was revealed in the sequences of RNAs 1 and 2: two sequence variants were assembled for RNA1 (97.0% nt identity; 99.3% aa identity in pair-wise comparisons), and two sequence variants were assembled for RNA2 (94.5% nt identity; 100% aa identity in pair-wise comparisons). These sequence variants were present in all three samples (Supplementary Table S2). No genetic diversity was found for RNA3 in all three samples of GaCLIdV (see Supplementary Table S2). Our attempts to acquire the 5′ and 3′ termini of the GaCLIdV genome segments were only partially successful, providing the 5′ and 3′ termini for GaCLIdV RNA1 of variant 1. Interestingly, the 5′-terminal stretch of 19 nucleotides (5′-GCACAAAGACCCTCTATAC-3′) of RNA1 was almost perfectly complementary to an 18 nt stretch (5′-GTATAGAGGTCTTTGTGC-3′) at the 3′ terminus of RNA1. Concomitantly, the nine terminal nucleotides of each RNA1 terminus were also highly similar to the genome segment termini reported for phenuiviruses [7]. Hence, the sequences of GaCLIdV deposited in GenBank under accession numbers PX111645 (variant 1, RNA1), PX111646 (variant 2, RNA1), PX111647 (variant A, RNA2), PX111648 (variant B, RNA2), and PX111649 (RNA3) represent complete or nearly complete genome segments.

**Figure 2:**
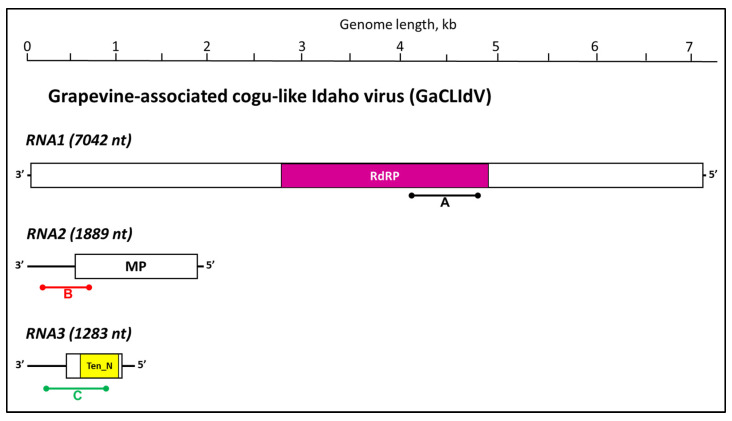
Schematic representation of the three grapevine-associated cogu-like Idaho virus (GaCLIdV) RNA genome components visualized as negative RNA strands. The open reading frames are represented as rectangles, with conserved protein domains designated with colors: RdRP = RNA-dependent RNA polymerase, Ten_N = tenui- and phlebovirus nucleocapsid domain. MP designates “movement protein” identified based on sequence similarity to the P2 protein of other cogu-like viruses. Three primer pairs specific to RNAs 1–3 were used for confirmation of GaCLIdV presence in the grapevine samples, which produced the PCR products A (black line), B (red line), and C (green line) shown under the genome diagrams.

The complete sequence of RNA1 was found to be 7042 nt long, the nearly complete sequence of RNA2 appeared to be 1889 nt long, and the nearly complete sequence of RNA3 resulted to be 1283 nt long. Each of the three RNA segments of GaCLIdV spanned a single open reading frame (ORF) encoding protein products exhibiting homology to bunyavirus RdRP (ORF1), movement proteins (MPs) of cogu-like viruses (ORF2), and nucleocapsids of tenui- and phleboviruses (Ten_N; ORF3), respectively (Figure 2). The sizes of the three genome segments of GaCLIdV and of the encoded proteins, 2313 amino acids (aa; L protein/RdRP), 393 aa (MP), and 260 aa (NP), were similar to those of the genome segments and proteins of switchgrass phenui-like virus 1 (SgPhLV-1; GenBank accessions PP996022–PP996024) and Laurel Lake virus (LLV) [29]. The overall genome organization of GaCLIdV was found especially similar to that of SgPhLV-1, with pair-wise comparisons of the virus-encoded proteins demonstrating between 40.1 and 49.7% identity levels (Table 2). The higher identity level between the RNA2-encoded MPs of GaCLIdV and SgPhLV-1 (49.7%) may reflect functional similarity in a plant host as opposed to the RNA2-encoded protein of LLV (only 27.7%), which was identified in ticks, an arthropod host [29].

### 3.2. Prevalence of GaCLIdV in Wine Grapes in Southwestern Idaho and in Eastern Oregon

**Figure 3:**
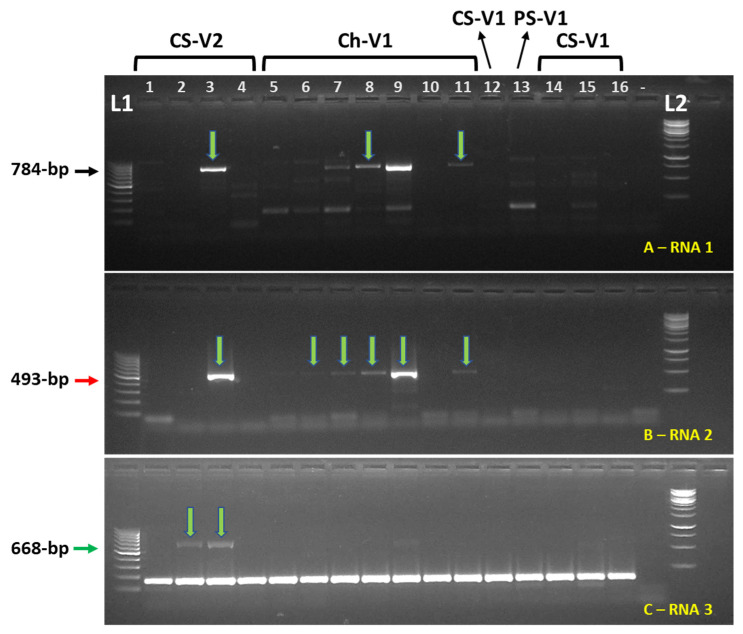
An example of the screening of 16 grapevine samples collected in September 2023 from two vineyards, V1 and V2, in Canyon County of Idaho for analyzing the presence of the grapevine-associated cogu-like Idaho virus (GaCLIdV). Reverse transcription (RT)-PCR was conducted with primer pairs A (784 bp, black arrow), B (493 bp, red arrow), and C (668 bp, green arrow) targeting three separate genome segments of GaCLIdV, see Figure 2 for details. The PCR products were separated in a 1% agarose gel and visualized under UV light after ethidium bromide staining. Lanes representing individual plants are numbered; “-“ designates the negative control (ddH_2_O). Select PCR bands marked with vertical arrows were Sanger-sequenced and confirmed to represent GaCLIdV sequences. Grapevine cultivar designations: CS = Cabernet Sauvignon; Ch = Chardonnay; PS = Petite Sirah.

Of the two grapevine samples collected in October 2018 and the two obtained in September 2020 in the old ‘Chardonnay’ block of vineyard V1 (see Figure 1), three were found to contain GaCLIdV by HTS (Table 1). In September 2021, three ‘Chardonnay’ samples collected in the same block of vineyard V1 were also found GaCLIdV-positive (Table 3); the confirmation of virus infection was based on the Sanger sequencing data from the amplified RT-PCR products. To determine if GaCLIdV could be found in other wine grape cultivars and in other vineyards, samples collected in 2023 and 2024 were analyzed by RT-PCR for GaCLIdV presence using primers specific to all three genome components. In total, for the entire period of 2020 to 2024, out of 140 tested samples, 27 samples from six wine grape cultivars grown in five different vineyards (V1 to V5) in Canyon County of Idaho and in one vineyard (V6) in Malheur County of Oregon were found GaCLIdV-positive (Table 3). The positive status was assumed if at least one primer pair specific to any of the three genome segments of the virus produced the PCR band of the expected size, and this product was confirmed to be GaCLIdV-specific after Sanger sequencing. The primer pair B specific to RNA2 encoding the putative MP protein produced the highest number of RT-PCR positives, i.e., 23 out of 27 (Table 3), indicating that this genome segment may be a good target for the development of a GaCLIdV diagnostic system. Interestingly, only a handful of GaCLIdV-positive samples, 5 out of 27, produced GaCLIdV-specific PCR bands with all three primer pairs, A to C, targeting all three different genomic segments (Table 3; Figure 3). This may suggest some genetic diversity in the genome sequences of GaCLIdV isolates in vineyards, requiring the identification of the most conserved genome regions and the optimization of the RT-PCR protocols.

In 2023, some of the samples were collected for an unrelated berry quality study, and thus a number of healthy-looking ‘Merlot’ leaf samples were acquired from vineyard V4 (Table 3). Interestingly, five ‘Merlot’ samples were found GaCLIdV-positive by RT-PCR, of which four came from asymptomatic and healthy-looking plants. This fact suggests the lack of involvement of GaCLIdV in symptom development, at least in the cultivar ‘Merlot’.

### 3.3. Phylogeny of the New Grapevine Cogu-like Virus

**Figure 4:**
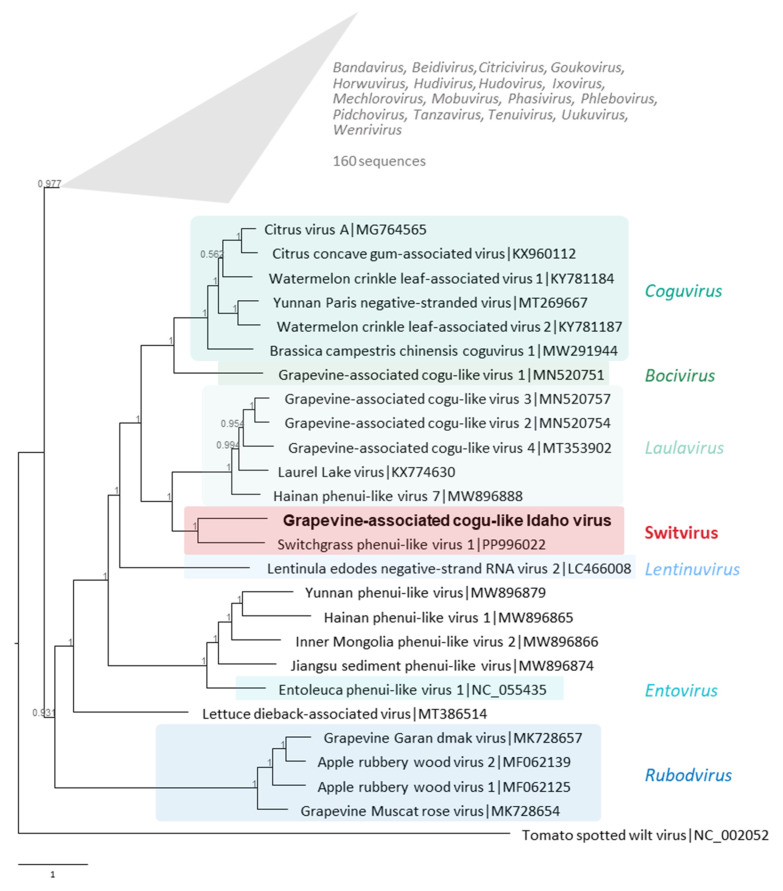
Phylogenetic analysis of the grapevine-associated cogu-like Idaho virus (GaCLIdV) full-length replicase proteins of members of the family *Phenuiviridae*. A maximum likelihood phylogenetic tree was inferred from the alignment of the replicase amino acid sequences of representative viruses of the family *Phenuiviridae* (see the list of sequences in Supplementary Table S1), using IQtree2 with ModelFinder to choose the best fit model and branch support estimated using aBayes test. GaCLIdV is highlighted in bold font. The proposed new genus, tentatively named Switvirus and comprising GaCLIdV, is highlighted in red, while the currently recognized *Phenuiviridae* genera used to build the phylogeny are indicated in shades of blue and green if visible and in grey if in the collapsed clade. The tree was rooted using the replicase of tomato spotted wilt virus as an outgroup, with the root arbitrarily placed on the edge connecting the ingroup and the outgroup. The original, fully annotated tree is available in Supplementary Figure S1. The scale bar shows the number of substitutions per site. The collapsed clade is not to scale.

Based on L-protein/replicase phylogeny, GaCLIdV was placed in a distinct clade close to the recently described switchgrass phenui-like virus 1 (SgPhLV-1; Figure 4). This clade is the closest to the genus *Laulavirus* comprising at least three cogu-like viruses associated with wine grapes (see Figure 4), although distinct from it based on long branch lengths and the high bootstrap value at the node separating this new GaCLIdV/SgPhLV-1 lineage from the *Laulavirus* lineage (Figure 4). Interestingly, the RdRP of GaCLIdV was placed within a larger lineage including the genera *Bocivirus* and *Laulavirus*, with several virus species associated with grapevines (Figure 4). At the same time, the RdRP of GaCLIdV was clearly placed separately from the genus *Rubodvirus*, which comprises two virus species found in grapevines (Figure 4; [3]).

## 4. Discussion

Deep sequencing has proven very useful in identifying viruses associated with main disease syndromes in grapevine and, as a side bonus, in the discovery of new viruses in *Vitis* spp. [30,31]. Here, we applied HTS to dissect the possible causes of the decline observed in an old ‘Chardonnay’ block from a commercial vineyard in Canyon County of Idaho. A new tri-partite virus, GaCLIdV, with (-) ssRNA genome (Figure 2), was discovered in these ‘Chardonnay’ vines exhibiting decline symptoms. The original samples collected in 2018 (18RB02) and 2020 (RB09 and RB12) were selected from ‘Chardonnay’ plants exhibiting leaf chlorosis, yellowing, and leaf necrosis (Figure 1). These same ‘Chardonnay’ samples were found to contain several other viruses, i.e., GLRaV-3, GVA, GVB, GVF, GRVFV, GaTLV, and endornaviruses (see [13,14,16]); hence, the role of GaCLIdV in the expression of any of the symptoms observed remains unclear. The samples collected from red- and white-berried cultivars in 2020, 2021, 2023, and 2024 (Table 3) were mostly selected based on various symptoms visible in the foliage of the wine grapes just before harvesting, mainly interveinal reddening in red-berried cultivars and leaf rolling and chlorosis in white-berried cultivars. Nevertheless, four of the five ‘Merlot’ samples from vineyard V4 collected in 2023 (Table 3) were asymptomatic, indicating no role of GaCLIdV in symptom expression in this cultivar. Based on the currently available data, no detrimental effects of GaCLIdV can be established in wine grapes.

The fact that GaCLIdV was found in five grapevine cultivars and in six vineyards operated by different owners indicates that the virus may be widely distributed across the two considered counties in Idaho and Oregon in the Snake River Valley AVA. Limited surveys of wine grapes in the Snake River AVA conducted during the four seasons of 2020, 2021, 2023, and 2024 suggested GaCLIdV prevalence at just under 20% (see Table 3). In the absence of GaCLIdV-specific testing in nurseries supplying planting material to growers in Idaho and Oregon, the spread of the virus may be via contaminated plants. Grafting was demonstrated previously as the only mode of transmission of two grapevine viruses from the genus *Rubodvirus* [3]. While no information is available on vector organisms involved in transmission of other cogu-like viruses in plants, their association with fungi found in vineyards [5,6] may potentially indicate a fungal vector. On the other hand, phenuivirids from the genus *Tenuivirus* are transmitted by planthoppers or leafhoppers in a circulative and propagative manner [8,32,33], while multiple genera of phenuiviruses infect or are transmitted by arthropods, insects, or ticks [7]. Additional experiments will be required to determine the vector transmissibility of GaCLIdV.

The latest ICTV update of the taxonomy of phenuiviruses lists 23 genera and 159 species in the family *Phenuiviridae*, with 6 genera, *Rubodvirus*, *Entovirus*, *Lentinuvirus*, *Bocivirus*, and *Coguvirus*, belonging to a monophyletic lineage comprising cogu-like viruses grouped together based on RdRP phylogeny [7]. Phylogenetically, the GaCLIdV replicase was placed inside this cogu-like lineage of phenuiviruses (Figure 4), close to SgPhLV-1 replicase, but the topology of the clades inside the cogu-like lineage suggests the separation of the GaCLIdV/SgPhLV-1 branch from other branches representing phenuiviruses, which is enough for designating a new genus of phenuiviruses. No publications could be found associated with SgPhLV-1 sequences in GenBank databases; however, the limited information in the sequence descriptions indicates the plant origin of SgPhLV-1, isolated from switchgrass (*Panicum virgatum*). Given the distinct position of GaCLIdV and SgPhLV-1 in the phylogenetic tree of phenuiviruses, we propose to assign them to a new genus with the provisional name Switvirus (Figure 4). This genus name was derived from the host name switchgrass, a host of the first virus, SgPhLV-1, discovered in this genus. Currently, this new genus includes two tri-partite viruses with a (-) ssRNA genome, SgPhLV-1 and GaCLIdV.

## 5. Conclusions

A new (-) ssRNA virus, the grapevine-associated cogu-like Idaho virus (GaCLIdV), was discovered in wine grapes in Idaho and Oregon, USA, by HTS and subsequent validation and confirmation of the virus presence by conventional RT-PCR and Sanger sequencing. Surveys of the grapevines from two counties in Idaho and Oregon indicated the presence of GaCLIdV in several grapevine cultivars and in multiple vineyards. Phylogenetic analysis of the RdRP domain of GaCLIdV designated this virus as a new species of the proposed genus Switvirus, family *Phenuiviridae*. The contribution of GaCLIdV to the expression of decline and leafroll symptoms observed in some of the sampled vines remains to be elucidated.

## Figures and Tables

**Table 1:** Summary of the high-throughput sequencing (HTS) outputs for the three ‘Chardonnay’ samples collected in 2018 and 2020. Raw reads were adapter- and quality trimmed. Clean read pairs were then mapped to the *Vitis vinifera* reference genome. Contigs were assembled from the unmapped read pairs.

Sample ID	Read Pairs	Cleaned Read Pairs	Unmapped Read Pairs	Contigs > 1000 nt
18RB02	20,840,871	19,368,940	6,340,098	22,692
RB09	15,095,042	14,203,522	1,925,857	2295
RB12	20,279,532	19,116,250	1,763,090	2327

**Table 2:** Pair-wise comparisons of nucleotide (nt) and protein sequences (aa) for grapevine-associated cogu-like Idaho virus (GaCLIdV) and two other phenuiviruses, switchgrass phenui-like virus 1 (SgPhLV-1) and Laurel Lake virus (LLV). L = L-protein; MP = movement protein; CP = capsid protein.

	SgPhLV-1		LLV	
	nt	aa	nt	aa
RNA1/L(RdRP)	NS ^a^	40.6%	NS	36.3%
RNA2/MP	NS	49.7%	NS	27.7%
RNA3/CP	NS	40.1%	NS	34.9%

^a^ NS = not significant.

**Table 3:** Pair-wise comparisons of partial nucleotide (nt) sequences generated by RT-PCR against the HTS sequences for grapevine-associated cogu-like Idaho virus (GaCLIdV) RNAs 1–3. In total, 27 grapevine samples collected from six vineyards (V1–V6) out of 140 tested in the 2020–2024 seasons were found GaCLIdV-positive. Cab. Franc = Cabernet Franc, Cab. Sauv. = Cabernet Sauvignon.

	Cultivar	Vineyard	RNA1_RdRP		RNA2_MP		RNA3_NP	
			nt identity to RNA1, %	GenBank Acc. Number	nt identity to RNA2, %	GenBank Acc. Number	nt identity to RNA3, %	GenBank Acc. Number
2020	Chardonnay	V1	99.6%	PX114093	100.0%	PX124612	99.7%	PX123833
	Chardonnay	V1	99.8%	PX114094	88.7%	PX124613	100.0%	PX123834
2021	Chardonnay	V1	NB ^a^	-	NB	-	98.0%	PX123835
	Chardonnay	V1	100.0%	PX114095	bad seq	-	NB	-
	Chardonnay	V1	99.9%	PX114096	91.2%	PX124614	bad seq	-
2023	Cab. Franc	V3	99.4%	PX114097	NB	-	NB	-
	Cab. Sauv.	V2	NB	-	NB	-	99.1%	PX123836
	Cab. Sauv.	V2	97.4%	PX114098	99.8%	PX124615	99.8%	PX123837
	Chardonnay	V1	bad seq	-	80.7%	PX124616	bad seq	-
	Chardonnay	V1	bad seq	-	92.5%	PX124617	NB	-
	Chardonnay	V1	98.0%	PX114099	91.2%	PX124618	NB	-
	Chardonnay	V1	bad seq	-	90.8%	PX124619	98.7%	PX123838
	Chardonnay	V1	100.0%	PX114100	99.5%	PX124620	NB	-
	Merlot	V4	96.6%	PX114101	NB	-	NB	-
	Merlot	V4	NB	-	99.8%	PX124621	NB	-
	Merlot	V4	99.9%	PX114102	99.8%	PX124622	NB	-
	Merlot	V4	89.9%	PX114103	99.6%	PX123845	NB	-
	Merlot	V4	100.0%	PX114104	91.7%	PX124623	NB	-
2024	Merlot	V3	97.5%	PX114105	99.8%	PX124624	99.8%	PX123839
	Riesling	V3	NB	-	99.8%	PX124625	NB	-
	Cab. Franc	V5	NB	-	100.0%	PX124626	100.0%	PX123840
	Tempranillo	V5	99.8%	PX114106	88.8%	PX124627	99.8%	PX123841
	Tempranillo	V5	NB	-	100.0%	PX124628	100.0%	PX123842
	Tempranillo	V5	bad seq	-	94.5%	PX124629	bad seq	-
	Chardonnay	V1	bad seq	-	91.1%	PX124630	97.2%	PX123843
	Chardonnay	V1	NB	-	91.3%	PX124631	100.0%	PX123844
	Tempranillo	V6	NB	-	98.4%	PX123846	NB	-

^a^ NB = no band.

## Data Availability

The sequences of the GaCLIdV genome segments 1, 2, and 3 are available in GenBank under the accession numbers PX111645 (variant 1, RNA1), PX111646 (variant 2, RNA1), PX111647 (variant A, RNA2), PX111648 (variant B, RNA2), and PX111649 (RNA3); the partial sequences from the field samples are available in GenBank under the accession numbers PX114093-PX114106 (RNA1), PX124612-PX124631, PX123845, PX123846 (RNA2), and PX123833-PX123844 (RNA3). The raw sequence data were deposited in the NCBI Sequence Read Archive (SRA) under BioProject number PRJNA931750.

## Supplementary Materials

The following supporting information can be downloaded at: https://www.mdpi.com/article/10.3390/v17091175/s1, Figure S1: Phylogenetic analysis of the grapevine-associated cogu-like Idaho virus (GaCLIdV) full-length replicase proteins of members of the family *Phenuiviridae*; Table S1: Primers used to confirm and validate the presence of three grapevine-associated cogu-like Idaho virus (GaCLIdV) genome segments in grapevine tissues; Table S2: Number of individual reads aligning to each cogu-like virus variant RNA1 in each sample analyzed.
