# Supplementary figures and images for: A Novel Cogu-like Virus Identified in Wine Grapes
Source: Viruses. 2025 Aug 28;17(9):1175. doi: 10.3390/v17091175 (PMC12474454; doi:10.3390/v17091175)

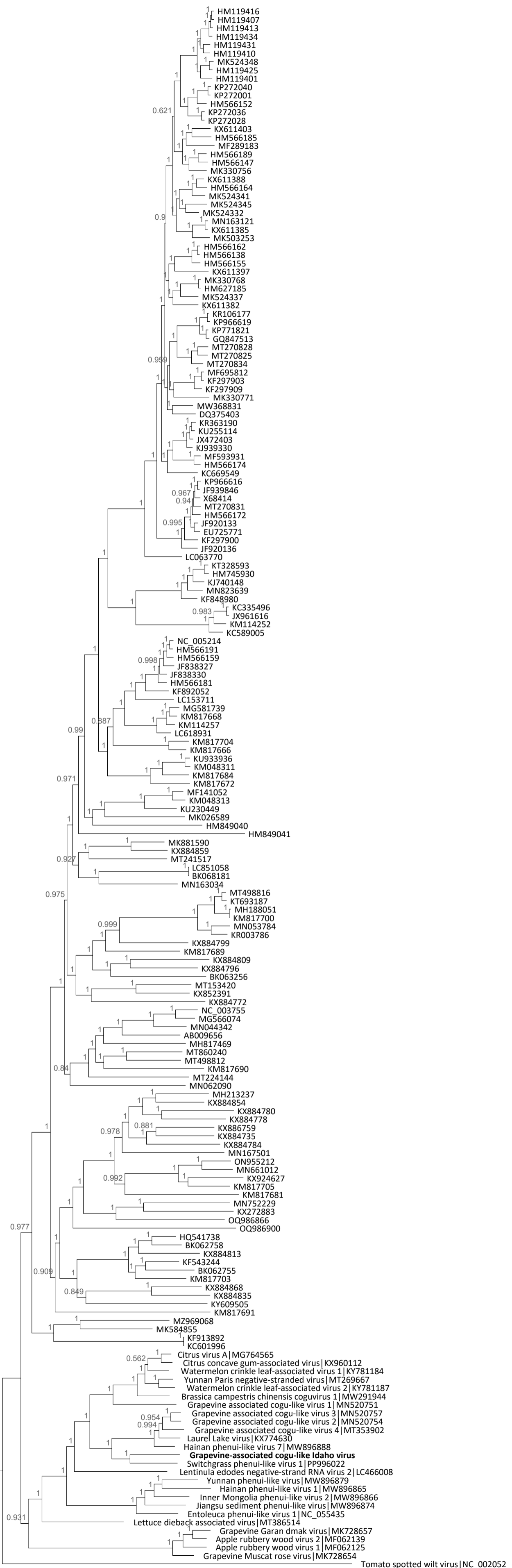

Supplement: Supplementary file 1 [file viruses-17-01175-s001.zip › Supp_FigS1_v2.pdf]
